# International Students’ Motivation to Study Abroad: An Empirical Study Based on Expectancy-Value Theory and Self-Determination Theory

**DOI:** 10.3389/fpsyg.2022.841122

**Published:** 2022-03-23

**Authors:** Yun Yue, Jinjin Lu

**Affiliations:** ^1^School of Education, Torrens University Australia, Sydney, NSW, Australia; ^2^College of Humanities and Social Sciences, Chaoyang University of Technology, Taichung, Taiwan; ^3^School of Education, University of Tasmania, Hobart, TAS, Australia

**Keywords:** international student mobility, expectancy-value theory, self-determination theory, push-pull theory, motivation

## Abstract

Push-pull theory, consumer decision-making models and rational choice theory are commonly used to explain international student mobility (ISM). Despite their merits, the individual’s motivation to study abroad is ignored. Based on two motivation theories—expectancy-value theory (EVT) and self-determination theory (SDT), this study examines whether students’ intention to study abroad originates from the students themselves or compromises social pressure and how the external factors defined in push-pull theory work with these motivations to affect their decision-making. A quantitative study was conducted with a sample size of 736 international students in China. The findings show that the decision to study abroad is a highly subjective and intrinsically driven behavior in which realizing one’s self-worth or fulfilling one’s purpose of life plays the most significant role. Making a decision needs a “cost-benefits calculus,” but the utility value of study abroad was positioned behind attainment value and identified motivation. The heterogeneity of international students’ motivation was also differentiated by both their gender and their parents’ educational backgrounds.

## Introduction

The push-pull theory has been widely recognized as one of the most valuable frameworks to theoretically interpret international student mobility (ISM) in the literature. Since McMahon (1992) first introduced the theory in ISM by examining international students from 18 developing countries during the 1960s and 1970s, there has been an increase in ISM research based on this theory. A typical one is Altbach (2004) study in which the push factors as relating to the students’ home countries’ limited space or enormous pressure to compete for higher education; a lack of high-quality education institutions; the availability of the specific courses that students want to study; discriminatory university admissions policies; and political or other forms of repression at home. Meanwhile, the pull factors include reputable academic institutions and degrees, the possible strengthening of employability after graduation, job opportunities in the host country, opportunities for relocation to the host country, targeted efforts to share knowledge or cultural ties with the home country, the availability of scholarships or other financial aid and marketing activities. Although the push-pull theory is recognized as one of the classic frameworks in the ISM field, it has been challenged by an argument that it pays too much attention to the macro or external factors and too little to the micro or personal factors which influence transnational mobility (De Haas, 2009). Therefore, a modified push-pull theory was developed by considering personal factors, including personal advancement and career development (e.g., Bamber, 2014), the development of intercultural awareness (e.g., Langley and Breese, 2005), family influence (e.g., Pope et al., 2014), access to local community networking in the receiving country (Sivakumaran et al., 2013), cost of living (Shanka et al., 2005), and escape from stressful situations or day-to-day life (Forsey et al., 2012). Although these personal factors help to broaden the cognitive boundary of push-pull theory, Lauermann (2015) argued that the identified personal factors in the modified push-pull theory are perceived as “concrete” (p. 197) factors rather than underlying intrinsic motives.

Besides, another two theoretical structures widely used in the literature to interpret the individual factors influencing international students’ decision to study abroad are: *consumer decision-making model* (CDM) and *rational choice theory* (RCT). CDM is derived from consumer behavior theory, in which making a purchasing decision is regarded as the outcome of consumer behavior. With CDM, a purchasing process consists of five stages: need recognition, information search, evaluation of alternatives, purchase and post-purchase evaluation (Oliveira and Soares, 2016). Then, the five-stage process was applied into the context of international education: need recognition/aspiration to study abroad, search for information, evaluation of alternatives, applying at HEI abroad, and confirmation (Haas and Terryn, 2019). In fact, the decision-making process is also a choice-making process. RCT assumes individuals as rational decision-makers who make choices by maximizing self-interests or utilities through a costs-benefits calculus (Eriksson, 2011). In the education context, benefits are identified to be associated with higher labor market or earnings returns (Browne, 2010) and the costs related to tuition fees or risk of failure (Gabay-Egozi et al., 2010). As long as the earnings potential outweighs the costs, students will participate in education (DesJardins and Toutkoushian, 2005).

Both CDM and RCT were rooted in the notion of the privatization and commodification of higher education in the context of neoliberalism, where higher education transformed from a public good into a commodity or a service to be bought, and the identity of the students changed from that of a learner to that of a consumer (Mowjee, 2013). In this sense, study abroad became more like a purchase behavior. However, treating educational decisions as purely economic behavior has brought many criticisms. Chloe (2019) and Mowed (2013) argue that the studying abroad decision-making is far more than a simple act driven by economic motivation but results from complex social factors acting on personal motives. For example, studying in a world-renowned higher education institution is regarded as a “symbol” or marker of the elite in East Asian culture. Thus, many families put massive pressure on their children (Waters, 2007), resulting in some students choosing to study abroad not driven by their own desire but succumbing to family pressure. Bamberger (2019) also claimed that ISM is not only the pursuit of “economic advantage” but “cosmopolitan capital” and “ethnic identity capital” (p. 1367) for some culturally and ethnically distinct groups. Marginson (2013) criticized the traditional understanding of international education as an “adjustment” to the host country norms and institutions, positing that it is a process of “self-formation” in which students reflectively manage their lives and shape their own changing identities. Advanced by the views of Marginson (2013), Tran (2015) considered ISM as a “becoming” process, in which international education “facilitates not only the redistribution of social class capital but, importantly the pursuit of the integrated forms of profession-advanced capital and migration-oriented capital” (p. 1286). All these studies are dedicated to discovering the motivations of international students studying abroad from various angles. However, the demographic heterogeneity of individuals was neglected despite identifying ethnical groups in some of the studies. More importantly, the measurement of the motivations to find out which one played a more significant role in the decision-making process than others and to what extent structural or external factors influenced the process are missed out in these studies. Hence, the study employed EVT and SDT as theoretical framework to structure the understanding. The two theories have been widely used in the literature to measure the motivation of ISM, but mostly focus on associating international students’ motivation with their adaptation and well-being in the host counties (e.g., Chirkov et al., 2007; Raczkoski et al., 2018; Ganotice et al., 2020).

## Theoretical Framework

### Expectancy-Value Theory

Most current research is based on the modern concept of EVT developed by Eccles and her research team (Eccles and Wigfield, 2002; Eccles, 2005; Wigfield et al., 2016). Expectancy-value theorists (Wigfield and Eccles, 2000) claim that “individual choice, persistence, and performance can be explained by their beliefs about how well they will do on the activity (expectancy) and the extent to which they value the activity (value)” (p. 68). *Expectancy for success* and *subjective task value* are two direct predictors of achievement choice. Eccles and Wigfield (2002) further proposed four types of value: *intrinsic value* (the inner enjoyment a person gains from performing the task), *attainment value* (the importance of success in undertaking a specific task), *utility value* (the usefulness of achieving a goal or completing a task) and *cost*. *Cost* also has four sub-components: *task effort cost* (how much time and effort are put into the task), *outside effort cost* (how much time and effort are spent on tasks other than the task of interest), *loss of valued alternatives* (LOVA, the loss of other options due to conducting the task), and *emotional cost* (negative emotions such as stress, worry and anxiety caused by engaging in the task) (Barron and Hulleman, 2014). Some studies recognize the *cost* as an independent component that parallels expectancy and value to understand the barriers better when individuals are unmotivated (e.g., Barron and Hulleman, 2014). Besides, Eccles emphasized EVT as a task-specific motivational model (Wigfield et al., 2016) because individuals may have completely distinct perceptions of expectancy, values and cost for different tasks. Hence, although the theory has been widely applied in areas of education, such as course selection (e.g., Nagy et al., 2008), homework (e.g., Trautwein et al., 2006), teacher education (e.g., Green, 2002), and educational psychology (e.g., Fan, 2011), the expectancy, value and cost attributed to various fields are distinct.

### Self-Determination Theory

Self-determination theory is another empirically-based motivational construct that is recommended to explain ISM’s intention (Chirkov et al., 2007; Lauermann, 2015). Based on Deci and Ryan (1985)’s intrinsic and extrinsic motivation, SDT has been developed into a motivation continuum to assess the degree to which an individual’s behavior is performed autonomously versus being controlled externally (Deci and Ryan, 2008). *Autonomous motivation* has two sub-categories: *intrinsic motivation* (the prototype of autonomous motivation, characterized by an individual engaging in a specific task simply because of his or her interest in it) and *internalized extrinsic motivation* (or *identified motivation*, a type of extrinsic motivation in nature but has been internalized or regulated into a self-willing belief). In the context of ISM, identified motivation is demonstrated when students are willing to study overseas because they believe that it can lead to better career development (Chirkov et al., 2007). *Controlled motivation* also has two sub-categories: *extrinsic regulation* (the individual’s behavior is motivated by external rewards or avoiding “concrete” punishment) and *introjected regulation* (the person is apt to implement the behavior to avoid feelings of guilt or shame or meet the expectations of others). When a person conducts an activity with controlled regulation, he or she is motivated by external factors or is under pressure caused by these external factors. It can explain why not all study abroad is self-determined, but due to pressure from family or other environmental factors.

### Research Objectives and Hypotheses Development

The study aims to achieve three research objectives. For each research objective, specific hypotheses were as follows.

RO 1: To examine to what extent the structural or external factors work with the motivations to determine their decision to study abroad.

Based on the criticism of the dominance of structural factors and ignorance of individuals’ internal motivations in studying abroad, the study argued that the decision to study abroad cannot be fully achieved by structural factors alone. Hence, a hypothesis was proposed as follow:

*H1: Structural factors alone are less effective in predicting intention to study abroad than the combination of structural and motivational factors.*

RO 2: To identify the fundamental driving forces of study abroad by measuring EVT and SDT elements.

Expectancy-value theory has been recently suggested to explain ISM (Lauermann, 2015), but it has not been used much. Raczkoski et al. (2018)’s study would be one of the few undertaken to find out students’ motivations at the College of Agricultural Sciences and Natural Resources (CASNR) to enroll in a short-term study-abroad course before graduation. Findings showed that intrinsic value and expectancy were the two strongest factors, while outside effort cost was the weakest factor related to the students’ motivation to take the course abroad. Given the scarcity of relevant research in the literature, we hypothesized, based on Raczkoski et al.’s (2018) study, that

*H2: Intrinsic value and expectancy were the two strongest predictive factors to the intention of study abroad.*

*H3: Outside effort cost was the weakest factor related to the students’ intentions to study abroad.*

SDT, in the existing literature, mainly focuses on examining the association between international students’ decision to study abroad, subjective well-being, and social adaptation and acculturation (e.g., Chirkov et al., 2007; Ganotice et al., 2020). Almost all of the findings support the argument that international students who are more self-determined in their decision to study abroad have higher autonomy, better academic performance, higher subjective well-being, and higher acculturation when they study in the host country. Given limited research examining the relationship between the intention of study abroad and autonomous and controlled motivation, it is unclear whether autonomous motivation or controlled motivation has a more significant predictive effect on study abroad intention. As such, we leave this as an exploratory research objective.

RO 3: To determine the differences in EVT and SDT to study abroad by genders, ages, and parents’ educational backgrounds.

It is documented in the literature that individuals’ intentions to study abroad are different by their genders, ages, and parents’ educational backgrounds. While the gender gap in study abroad that females are more likely to study abroad than males has been widely recognized, there is no consensus on the differences in intention to study abroad among males and females. Although some studies (e.g., Lindsay, 2014) claimed no significant differences in intentions to study abroad, most of studies (e.g., Salisbury et al., 2010; Hurst, 2018; Van Mol, 2021) agreed that women are more motivated to study abroad than men because of “a long-standing historical forms of gendered capital that equates women’s upward mobility and class reproduction with leaving home” (Hurst, 2018, p. 11). Also, older students are identified to be more hesitant to study abroad than younger ones (Pope et al., 2014) because they have more social obligations (e.g., full-time jobs) and family responsibilities (e.g., spouse and children). Studying abroad can be a difficult decision for them as they need to suspend their current jobs and leave home for a while. Given these difficulties, Kim and Goldstein (2005) found that older students have lower intentions to study abroad than youngers. Besides, parents’ educational backgrounds are determined to influence individuals’ intention to study abroad (Miller, 2008; Pope et al., 2014). Parents with higher education degrees tend to encourage their children to pursue tertiary education after completing secondary education and give more suggestions on their course selection (Salisbury et al., 2010). Also, those with well-educated parents usually come from higher-income families and attend better secondary schools, which can finance their enrollment and give them a desire to study abroad (Kim and Lawrence, 2021).

Despite the well-documented demographics influencing study abroad intentions, few scholars tried to explain the motivations behind the intention. For example, is the intention driven by the utility value of studying abroad or individuals’ intrinsic interests? Hence, we need EVT and SDT to theoretically decompose motivations so that the mechanism of intention can be well explained. As such, we leave this as an exploratory research objective as well.

## Materials and Methods

### Setting

The study took international students in China as a case. China has been the third-largest international student host country, followed by the United States and the United Kingdom since 2011 (ICEF, 2014). According to the Ministry of Education of the People’s Republic of China (MOE, 2019), in 2011, 292,611 international students enrolled in China’s higher education institutions. By 2018, the number increased to 492,185 (40.54% growth from 2011). The top five source countries are South Korea, Thailand, Pakistan, India, and the United States. The study was conducted at two universities in China: one in Beijing, and another is in Nanjing, the capital city of Jiangsu Province. Beijing and Jiangsu Province are the two regions with China’s largest and third-largest international students, with 80,786 and 45,778 students enrolled in 2018, respectively.

The present study is based on a retrospective design in which participants were asked to recall what factors affected their decisions to study in China. The retrospective design aimed to circumvent the concern about “fine-tuning” individuals’ views (Battle and Wigfield, 2003, p. 69) in response to future tasks (Hagemeier and Murawski, 2014).

### Participants and Procedure

The study employed a simple random sampling method to collect quantitative data. According to unpublished university statistics from the two universities, by 31st December 2018, 6,578 international students took degree courses at the two universities. By consent of the universities’ student offices, we assigned a number to each student from 0001 to 6578. With a confidence level of 95% and a confidence interval of 2, the sample size needed was calculated as 1,759. With an online random number generator, 1,759 numbers were then randomly selected. On 20th August 2019 and 7th September 2019, two rounds of invitation emails with a URL to access the questionnaire were sent out to the 1,759 students. The questionnaire was anonymous, and each completed questionnaire was sent back to the researchers’ administrative account without disclosing the students’ identities. After two rounds of invitations, a total of 742 responses was received. The response rate was 42%. After data cleaning, 736 responses were finalized. Please find the demographical features of respondents in Table 1.

**TABLE 1:** Sample demographics (*N* = 736).

		Numbers	Percentage
Gender	Male	471	64%
	Female	265	36%
Age	19–23	177	24%
	24–29	353	48%
	Above 30	206	28%
Regions of origin	East Asia/ASEAN	210	29%
	Africa	98	13%
	South Asia/West Asia	180	24%
	Central Asia/CIS countries/CEE	206	28%
	Others	42	6%
Degree currently in progress	Bachelor’s	287	39%
	Master’s	309	42%
	Ph.D.	140	19%
Parents’ education	one of the parents had a tertiary education degree	383	52%
	both parents had degrees in higher education	199	27%
	neither parent had ever studied at universities	154	21%

### Instrument

A seven-section survey questionnaire was designed to collect respondents’ demographic information and their perceptions of the extent to which certain factors affected their decisions to study abroad. Except for the section *About you—demographic information*, a nine-point Likert scale ranging from 1 for “strongly disagree” to 9 for “strongly agree” was used to rank respondents’ responses for each item.

The psychometric properties of the instrument were evaluated to ensure good reliability and validity before finalization. We analyzed the inter-item correlations for each scale and then submitted those with correlation coefficients > 0.20 to exploratory factor analysis using varimax rotation and principal-axis factoring (PAF). Factors were considered meaningful if they had an eigenvalue > 1. To maintain the final instrument, items needed to have a minimum factor loading of 0.40. The internal reliabilities of meaningful factors were estimated using Cronbach’s α. Items loading on a single factor with internal reliability coefficients of.70 or more were summed to form a scale score. Then, we evaluated construct validity of scales by seeing if they correlated in the directions we expected they would. For example, *Cost* scale would negatively correlate with the scales for *Intention to Study in China*, *External Factors*, *Expectancy for Success*, *Subjective Task Values*, *Autonomous Motivation* and *Controlled Motivation*. Other scales were expected to positively correlate one another. Please find the inter-scale correlation coefficients Table 2 below.

**TABLE 2:** Bivariate correlations between variables.

	Intention to study in China	External factors	Expectancy for success	The subjective task values	Cost	Autonomous motivation	Controlled motivation
Intention to study in China	−	0.21**	0.45**	0.35*	−0.45**	0.35*	0.39**
External factors		−	0.32**	0.19**	−0.22**	0.41**	0.23*
Expectancy for success			−	0.26*	−0.14*	0.32*	0.26*
The subjective task values				−	−0.16**	0.24**	0.31**
Cost					−	−0.19**	−0.24**
Autonomous motivation						−	0.35*
Controlled motivation							−

*p < 0.05, **p < 0.01.

Table 2 shows that *Cost* scale negatively correlated with the scales for *Intention to Study in China*, *External Factors*, *Expectancy for Success*, *Subjective Task Values*, *Autonomous Motivation* and *Controlled Motivation*, and other scales positively correlated one another. However, the correlations between the scales of *Subjective Task Values* and *External Factors*, *Cost* and *Expectancy for Success, Autonomous Motivation* and *Cost* were relatively low. The instrument was finalized as below:

#### About You—Demographic Information

This section was designed to collect participants’ demographic information, including gender, age, region of origin, an academic degree in progress and parents’ educational background.

#### Intention to Study in China

Three items were developed to assess the degree of their intention to study in China [e.g., “China was always my first choice for studying abroad,” “I might go to other countries if I had a choice” (reverse scored)]. The Cronbach’s α was 0.70.

#### External Factors

Based on the literature on push-pull factors, a 15-item subscale was designed to measure the extent to which external factors influenced the students’ choice of China as their study abroad destination (e.g., “I came to China because China has “rapid economic development,” “rich culture and long history,” and “the low crime rate”). The Cronbach’s α was 0.81.

#### Expectancy for Success

Three items were designed to assess international students’ beliefs about studying in China before their departure [e.g., “I believed I could study in China,” “studying in China would be a big challenge for me” (reverse scored)]. Cronbach’s α is 0.73.

#### The Subjective Task Value of the Studying-Abroad Scale

Modified *Valuing of Education Scale (VOE)* originally designed by Battle and Wigfield (2003) to examine people’s valuing of education, a 15-item scale was developed to fit in the context of international student mobility in China. It includes three value components: *interest* (four items, e.g., “I find the idea of studying China to be very appealing,” Cronbach’s α = 0.75), *attainment* (five items, e.g., “I feel that studying in China is a necessary part of what will make me feel good about myself in the future,” Cronbach’s α = 0.80) and *utility* (six items, e.g., “an overseas university degree is important to me because it will provide better job opportunities,” Cronbach’s α = 0.83). The overall Cronbach’s α is 0.83.

#### Cost of Study-Abroad Scale

The cost component is initially part of *VOE* but extracted to be an independent scale. A total of 10 items was developed to measure the cost associated with study abroad, including *task effort cost* (three items, e.g., “studying in China would not be worth it because I need to spend much money,” Cronbach’s α = 0.76), *outside effort cost* (two items, e.g., “it is time-consuming to learn Chinese in order to study in China,” Cronbach’s α = 0.71), *loss of value alternative* (two items, e.g., “I was worried that spending all the time studying in China would take time away from other activities I really wanted to do,” Cronbach’s α = 0.69) and *emotional cost* (three items, e.g., “I’d be embarrassed if I studied China and found out that my work was inferior to that of my peers,” Cronbach’s α = 0.74). The responses to this scale were reverse scored. The overall Cronbach’s α was 0.81.

#### Self-Regulation Questionnaire—Study Abroad

This 21-item scale was designed by Chirkov et al. (2007) specifically to measure to what extent autonomy played a role in international students’ decisions to study abroad. It has four subscales: *external regulation* (four items, Cronbach’s α = 0.83), *introjected regulation* (eight items, Cronbach’s α = 0.90), *identified regulation* (five items, Cronbach’s α = 0.88) and *intrinsic regulation* (four items, Cronbach’s α = 0.82). The overall Cronbach’s α was 0.86.

## Results

### Descriptive Analysis

Descriptive analyses were conducted to determine the differences in EVT and SDT to study abroad by genders, ages, and parents’ educational backgrounds (RO3).

Before any data analysis, we used Kolmogorov-Smirnov statistical test to examine sample normality. Results of data scanning indicated that all the variables in this study are not normally distributed as all the *p*-values are less than 0.05. Hence, alternative non-parametric statistic tests were used for data analysis. Mann–Whitney *U* tests were conducted to determine whether there were significant differences between genders in response to these scales. The results indicate that females had significantly higher intention (Mann–Whitney *U* = 12,411.5, *p* < 0.01, *r* = 0.15) and tended to be more autonomous (Mann–Whitney *U* = 12,094, *p* < 0.010, *r* = 0.15) than males did in their motivation to study abroad. However, men highly valued utility (Mann–Whitney *U* = 10,945, *p* < 0.000, *r* = 0.32) caused by study abroad (more than women), and they also considered there to be a higher LOVA cost (Mann–Whitney *U* = 11,040.5, *p* < 0.000, *r* = 0.22) and emotional cost (Mann–Whitney *U* = 12,150.5, *p* < 0.050, *r* = 0.30) (more significantly than women).

Kruskal–Wallis ANOVA (and Tukey *post hoc* test if necessary) were employed to test for significant differences in age group, regions of origin, academic degree in progress and parents’ educational background. The results showed that those whose parents had degrees in higher education had significantly higher intrinsic value (Chi-square *X*^2^ = 8.509, *p* < 0.010, η^2^ = 0.030) and autonomous motivation (Chi-square *X*^2^ = 7.838, *p* < 0.010, η^2^ = 0.023) but lower utility value (Chi-square *X*^2^ = 18.342, *p* < 0.000, η2 = 0.056) and controlled motivation (Chi-square *X*^2^ = 8.189, *p* < 0.050, η^2^ = 0.025) than whose parents do not have degree in higher education who have significantly higher controlled motivation in study abroad. There were no significant differences within age groups, regions of origin or academic degrees in progress.

### Hierarchical Multiple Regression Analysis

Hierarchical multiple regression analysis (Table 3) were conducted to examine to what extent the structural or external factors work with the motivations to determine their decision to study abroad (RO1) and to identify the fundamental driving forces of study abroad by measuring EVT and SDT elements (RO2).

**TABLE 3:** Unstandardized β-coefficients from hierarchical multiple regression (HMR) analyses.

	Model I	Model II	Model III	Model IV
External factors	0.736***	0.725***	0.798**	0.607***
Expectancy or belief		0.448*		0.304*
Intrinsic value		0.805**		0.723**
Attainment value		0.897***		0.798***
Utility value		0.799***		0.654**
Task effort cost		−0.687**		−0.479**
Outside effort cost		−0.395**		−0.298*
Loss of value alternative (LOVA)		−0.614**		−0.416**
Emotional cost		−0.476**		−0.351*
Intrinsic motivation			0.889**	0.687***
Identified motivation			0.958**	0.751**.
Extrinsic regulation			0.734*	0.587**
Introjected regulation			0.679**	0.527*
**Controlled Variables**				
– Gender	0.289*	0.132**	0.178*	0.127*
– Age groups	0.109	0.098	0.126	0.079*
– Regions of origin	0.097	0.045	0.075	0.037
– Degree in progress	0.030	0.026	0.068	0.021
– Parents’ educational level	0.108*	0.076*	0.096	0.064*
*R* ^2^	0.156	0.272	0.249	0.410
*R*^2^ change		0.116	0.093	0.138

*p < 0.05, **p < 0.01, ***p < 0.001.

A three-level HMR was conducted to explore further the predictive effects of variables derived by EVT and SDT on students’ intentions to study abroad. In the regression model, students’ intention to study abroad was the dependent variable. Demographic factors, including gender (female = 0, male = 1), age (19–23 years old = 0, 24–29 years old = 1 and above 30 = 2), regions of origin (East Asia/ASEAN = 0, Africa = 1, West Asia/South Asia = 2 and Middle Asia/CIS/CEE = 3), degree in progress (bachelor’s degree = 0, master’s degree = 1 and doctoral degree = 2), parents’ education background (neither with degrees in higher education = 0, one of the parents with degree in higher education = 1, and both with degrees in higher education = 2) were controlled variables. External factor Expectancy for success, subjective task value, cost and external factors were entered as independent variables at the first-level regression. Autonomous motivation and controlled motivation derived from SDT were entered as independent variables at the second level.

### Model I: External Factors Regression

In Model I, intention to study abroad as a dependent variable was entered into the model. Demographic factors as controlled variables were entered at the first step. External factors were entered as independent variables at the second step. The results show that “external factors” is a significant predictor of intention to study abroad, and when students’ perceptions of external factors increased by one unit, their intention to study abroad was expected to increase by 0.736 units. The amount of outcome variance explained by the external factors variable was 0.156.

### Model II: EVT Regression

In Model II, EVT’s variables, including expectancy, intrinsic value, attainment value, utility value, task effort cost, outside effort cost, LOVA, and emotional cost were entered into Module I. The results showed that all the independent variables were statistically significant predictors of students’ intentions to study abroad. Among them, attainment value had the largest predictive value (β = 0.897), which means that when students’ attainment value scores increased by one unit, their intention to study abroad increased by 0.897 units. The second-and the third-largest positive predictors are intrinsic value (β = 0.805) and utility value (β = 0.799), followed by the external factors (β = 0.725). By contrast, costs were significantly negatively predictive of students’ intention to study abroad. Task effort cost (β = −0.687) and LOVA (β = −0.614) are the largest and second-largest negative predictors. As students’ perceptions of task effort cost and LOVA increased by one unit, their intention to study abroad was expected to decrease by 0.687 and 0.614 units, respectively. Emotional cost (β = −0.476) and outside effort cost (β = −0.395) have a relatively lower negative predictive effect on the students’ intention. Expectancy (β = −0.448) is the second-smallest predictors. The amount of outcome variance explained increased to 0.272 after EVT variables were added.

### Module III: SDT Regression

In this Module, external factors and four SDT variables—intrinsic motivation, identified motivation, extrinsic regulation, and introjected regulation are included as independent variables. The findings showed that all four variables were positive predictors to the intention of studying abroad. The two autonomous motivation—intrinsic (β = 0.889) and identified motivation (β = 0.958) have higher predictive effects than the two controlled motivation—extrinsic (β = 0.734) and introjected regulation (β = 0.679). From Model I, the amount of outcome variance explained increased to 0.249.

### Model IV: Expectancy-Value Theory and Self-Determination Theory Regression

Self-determination theory and EVT variables were included in this extended regression model, and the amount of outcome variance explained increased to 0.410. In this model, attainment (β = 0.798) and identified motivation (β = 0.751) were the top two most significant positive predictors. When students’ perceptions of attainment values and their identified motivation increased by one unit, their intention of studying abroad increased 0.789 units and 0.751 units. Intrinsic value (β = 0.723) and intrinsic motivation (β = 0.687) is the third and fourth-largest predictors, followed by utility value (β = 0.654) and external factors (β = 0.607). The two controlled motivation—extrinsic regulation (β = 0.587) and introjected regulation (β = 0.527) ranked behind them. Within cost group, task effort (β = −0.479) and LOVA (β = −0.416) were two larger negative predictors, while emotional cost (β = −0.351) and outside effort cost (β = −0.298) are smaller negative predictors. Expectancy (β = 0.304) was the second-smallest predictor just above outside effort cost.

## Discussion

Based on the theoretical framework of EVT and SDT, this study aimed to determine the fundamental driving forces behind international students’ intention to study abroad by quantifying and ranking their motivations. One of the contributions of this study is that it empirically confirmed that external factors alone were insufficient to lead to the decision to study abroad, absent a student’s connecting with and acting on personal motivation (RO1). Instead, the strongest predictors of participants’ intention to study abroad were found to be attainment value and identified motivation (RO2), which will be discussed in detail later in this section. Another contribution of the study is its consideration of the heterogeneity of international students’ motivations, which were differentiated by both their gender and their parents’ educational backgrounds (RO3).

RO 3: To determine the differences in EVT and SDT to study abroad by genders, ages, and parents’ educational backgrounds.

Firstly, female participants were found to have a greater intention and more autonomy in their decision-making relative to studying abroad than males, who appeared to be more hesitant than females. This finding supports previous studies, in which females were found to have a higher intention to study abroad than males (e.g., Salisbury et al., 2010; Hurst, 2018). Moreover, this study also empirically revealed that males tended to emphasize the utility value of studying abroad more than females. Males were also more likely to be impacted by the LOVA and emotional costs of studying abroad. Males were more likely to “weigh up various pros and cons for investing time in a host of activities” and “place study abroad at a lower priority” (Hurst, 2018, p. 12) if they perceived that study abroad offered no instrumental benefits—for example, career development—or if they valued other things as more important than studying abroad.

Surprisingly, negative emotions caused by studying abroad also had a more significantly negative impact on men than women, which contradicted the general perception that women have stronger avoidance motivation in the face of negative emotions (Deng et al., 2016). It was assumed that men considered negative experiences caused by studying abroad as a “failure.” To save face, they were more likely to employ an avoidance attitude toward study abroad when they encountered difficulties.

This study also found that participants with well-educated parents had significantly higher intrinsic value and autonomous motivations for studying abroad, while those from less-educated families had higher utility value and controlled motivations. Although previous studies (e.g., Boudarbat and Montmarquette, 2009; Kim and Lawrence, 2021) had identified the relationship between parents’ educational backgrounds and students’ study abroad decision-making, few studies have distinguished between the types of motivation to study abroad determined by different family backgrounds. These findings enrich the field of study by indicating that students from well-educated families were more likely to be driven by their interest or pleasure when deciding to study abroad. In contrast, those from less-educated families highly valued the instrumental usefulness of studying abroad and showed significantly higher non-autonomous motivation in decision-making.

Similar arguments can be found in the literature, but most come from studies that explored the relationship between academic motivation and socioeconomic status (SES). The findings of this study could empirically support the argument that people’s SES can have a fundamental impact on their academic motivation (Deci and Ryan, 2009) if parents’ educational backgrounds can be regarded as one of the indicators of SES. They also support the findings that lower SES students had lower scores for intrinsic motivation and identified regulation and higher scores for external regulation than central and higher SES students in their academic motivation (Manganelli et al., 2021).

RO 1: To examine to what extent the structural or external factors work with the motivations to determine their decision to study abroad.

In a set of HRM, external factors and the components of EVT and SDT were included to determine which one played a greater role in the decision-making process and to what extent external factors influenced the process. The claim of Eccles (2009), cited by Wang and Degol (2013, p. 306–307) of the behavior choice being “influenced by a relative within-person hierarchy of expectations for success and subjective task values across the set of options considered” provided a theoretical rationale to adopt this approach.

Through comparison of four regression models, external factors were found to have a relatively low explanation of the variance (Model I) before variables of EVT and SDT were added (Models II, III, and IV). This finding empirically supported the H1 and arguments of Hadler (2006) and De Haas (2009), who found that external factors can define student mobiles but do not cause them, and they are only important in terms of their impact on individual motivation. The finding also supported H1.

RO 2: To identify the fundamental driving forces of study abroad by measuring EVT and SDT elements.

Attainment value and intrinsic values were identified by this study as the two strongest predictors of study abroad decision-making. In other words, individuals’ perceptions of the importance of studying abroad as befitting their identities and to what extent studying abroad could satisfy their inherent interests were two fundamental driving forces. The results partially support the H2. Utility value was a predictor behind the two values. The hierarchy ranking can be explained such that, even if studying abroad could lead to high utility value, if an individual did not consider it important for fulfilling his or her meaning of life or could not arouse any inherent interest, he or she was more likely to decide against studying abroad. More importantly, the findings empirically supported the criticism of CDM and RCT that the decision to study abroad was not a behavior dominantly driven by economic or instrumental benefits (e.g., higher educational returns) (DesJardins and Toutkoushian, 2005).

External factors were found to be predictors behind the three types of value, a finding that again confirmed that external factors are not the most determining driving force of an individual’s decision to study abroad. External factors were positioned between values and costs (Models II and IV), assuming that external factors work to help individuals judge the values and costs of studying abroad. This assumption attempted to break the “ontological dualism” (Piiroinen, 2014) between structure and agency in ISM literature by discussing the two in one discourse. However, it requires further empirical confirmation in future studies.

The element of costs was ordered behind values and external factors, and all were negatively predictive of the intention of study abroad as well as negatively associated with value. This positioning aligns with the EVT’s theoretical claim (Barron and Hulleman, 2014) that cost can be parallel to expectancy and value and works as an unmotivated force to prevent behavior choice. Also, the findings of that task effort cost had the highest score among the “cost” group, and outside effort cost had the lowest score within the “cost” group and all the variables, which supported H3. However, expectancy was found to be the second-lowest predictor in Model II and IV, which indicated that students might not believe that their effort would lead them to study abroad successfully. It is partially against H2 and contradicted Raczkoski et al. (2018)’s finding that expectancy is one of the two strongest predictive factors to the decision of studying abroad. However, some EVT theorists (Guo et al., 2015) argue that since an individual’s intentions are usually affected by the interaction between the two (values × expectancy), high scores of values can partially compensate for low expectancy. This study had high scores in values, so the low expectancy did not substantially influence people’s intention to study abroad. However, a further study, preferably qualitative research, is needed to have a future exploration here.

In Model IV, the outcome variance was increased after both EVT and SDT elements were added. It means that the combined variables of EVT and SDT are more predictive of students’ intention to study abroad than a single EVT theoretical framework. Although EVT and SDT are two theoretical frameworks and come from different theoretical perceptions with distinct intellectual roots (Wigfield and Eccles, 2000), they can complement each other. Compared with EVT, SDT is a theoretical construct concerned more with the quality of that motivation, i.e., the extent to which the behavior is from the heart (Deci and Ryan, 2008). In this model, identified motivation and intrinsic motivation had higher predictive effects than extrinsic regulation and introjected regulation on the participants’ intention, which means that autonomous motivations are more influential on individuals’ decision to study abroad than controlled motivations.

Furthermore, attainment value and identified motivation were higher than intrinsic value and intrinsic motivation. It means that despite an individual’s intrinsic interest significantly influencing their decision to study abroad, realizing one’s self-worth or fulfilling one’s sense of purpose is the most fundamental motivation for studying abroad, although the self-fulfilling belief might result from the internalization of extrinsic motivation.

## Conclusion and Limitations

In view of the dominance of research on structural factors and less attention to agency or motivational factors in the ISM, the study employed two motivation theories—EVT and SDT, by mean of a quantitative method, to examine to what extent motivational factors affect international students’ intention to study abroad. The structural or external factor was also included in determining its role in the motivational scheme. The study empirically supported the assumption in the literature that external factors alone are insufficient to lead to the decision without personal motivations. Besides, attainment value and identified motivation were identified as the two strongest determinants, which indicated that study abroad decision-making is a behavior autonomously driven by realizing one’s self-worth or fulfilling one’s sense of purpose. Utility value was positioned behind attainment value and identified motivation. It is an empirical refutation of the CDM and RCT’s claims that the decision to study abroad was not a behavior dominantly driven by instrumental benefits. The heterogeneity of international students’ motivation was differentiated by both their gender and their parents’ educational backgrounds. Female participants showed a greater intention and more autonomy in their decision-making than males. Those with well-educated parents had significantly higher intrinsic value and autonomous motivations and lower utility value and controlled motivations than those from less-educated families. In short, decision-making about studying abroad is more complex than expected, and it needs to be considered in a broader psychological and social context.

Despite some insights into the ISM provided, the study has some limitations. One of them was the weak correlations between some scales. Weak correlations could be caused by the non-linearity of the two variables, the small size of the sample, or the effects of discontinuous distributions, which may compromise the validity and generalizability of the results. Further studies are needed here for a strong justification. Another limitation is using single quantitative research. Qualitative research will be conducted in future studies to explore the findings in more detail. For example, it has been found that the attainment value is the most influential decision-making factor. Attainment value is a concept that is usually connected with the individual’s conception of their identity and ideals or their competence in a given domain. Hence, such questions as what the individual’s identity and ideals are, how they are formed and how they influence their perceptions of studying abroad can provide future research opportunities.

## Data Availability Statement

The original contributions presented in the study are included in the article/supplementary material, further inquiries can be directed to the corresponding author/s.

## Ethics Statement

Ethical review and approval were not required for this study on human participants in accordance with the local legislation and institutional requirements. Written informed consent from the participants was not required to participate in this study in accordance with the national legislation and the institutional requirements.

## Author Contributions

YY contributed to the conception and design of the study and wrote the first draft of the manuscript. JL conducted the statistical analysis, developing research objectives and hypotheses. Both authors contributed to manuscript proofreading and editing and approved the submitted version.

## Conflict of Interest

The authors declare that the research was conducted in the absence of any commercial or financial relationships that could be construed as a potential conflict of interest.

## Publisher’s Note

All claims expressed in this article are solely those of the authors and do not necessarily represent those of their affiliated organizations, or those of the publisher, the editors and the reviewers. Any product that may be evaluated in this article, or claim that may be made by its manufacturer, is not guaranteed or endorsed by the publisher.
